# Supplementary material for: Natural hybridization in heliconiine butterflies: the species boundary as a continuum
Source: BMC Evol Biol. 2007 Feb 23;7:28. doi: 10.1186/1471-2148-7-28 (PMC1821009; doi:10.1186/1471-2148-7-28)
Supplement: Additional File 1 — Hybrids between species of Heliconius and Eueides butterflies: a database. HTML file linking to database of all known wild-caught interspecific hybrid specimens in the Heliconiina, consisting of introductory text, a list of specimens, together with collection data and photographs of the specimens, and links to information about some artificial hybrids and mutants in the group. This is an edited copy of our online database of Heliconius hybrids [102]. To view database, download zip file and extract to a separate folder, then open index.html within that folder. [file 1471-2148-7-28-S1.zip › index.html]

Hybrids between species of Heliconius


# Hybrids between species of *Heliconius* and *Eueides* butterflies: a database


## by James Mallet, Walter Neukirchen and Mauricio Linares


© 1997-2006   
  
**Database of
natural *Heliconius* hybrids**


---

One of the best ways of showing evolutionary continuity
between species and geographic races is to demonstrate that hybridization still
occurs between closely related species. In Victorian times and early this century,
naturalists were very interested, like "stamp-collectors", in freaks of nature,
including rare hybrids between species. Between the 1930s and about 1980, there
was decreased interest about the peculiarities of nature, and increased emphasis
on the "fundamental" biological "realities" of animal species. (Hybridization
between plant species is so abundant and easily shown, of course, that this
rather myopic view of "pure", "good" species has never really caught on among
botanists). Widely used field guides from this period often omit pictures even
of common hybrids of birds and butterflies that can be seen in the wild, while
treating much rarer or even extinct species in the same book. Recently, however,
there has been renewed interest in all aspects of biodiversity, including that
within species, and it is possible to discern a return of interest in variants,
hybrids, and exceptions ("bad species") as well as the good species. In some
beautiful recent books, often thorough world treatments of particular groups
of butterflies and birds, hybridization between species is again becoming well-documented.

There has also been an impressive amount of recent
work on hybrid zones, but most of this work has concentrated, perhaps for obvious
reasons, on zones where hybrids are easily obtained, such as Henry Walter Bates'
(1863) pioneering work on natural hybridization in *Heliconius*, and
William Beebe's (1955) first experimental crosses; these are now considered
to be hybridization between geographic races of the same species). Arguably,
these studies contribute little to understanding speciation, because the forms
that interact have clearly *not* speciated.

Hybrids between species are much rarer: usually
less than one in a thousand individuals in a pair of hybridizing species are
recognizable hybrids, and often even fewer. What is not generally realized,
however, is that the fraction of all species that hybridize is high (Mallet
2005). A world-wide survey of birds has shown that around 9% of species hybridize
(Panov in Grant & Grant 1992), and in European butterflies including Hesperiidae,
the rate is about 12% of species (Guillaumin & Descimon 1976) - here species
are classified conservatively using the polytypic species concept, not the so-called
"phylogenetic concept", so hybridization between geographic forms is not considered
as interspecific hybridization, unless hybrids are very rare in the zone of
overlap. Some genera and higher groups have much higher rates, over 20% of species,
for example in the American warblers, the birds of paradise, and Darwin's finches
among the birds (Grant & Grant 1992). See also Mallet (2005) for a review
of natural hybridization in animals which surveys a number of groups, including
birds, mammals, as well as insects, and compares them to hybridization rates
in plants.

A somewhat related topic is the topic of hybrid
speciation. The speciation of taxa due to hybridization requires, of course,
the existence of ongoing natural hybridization documented here. Recent publications
provide conclusive evidence that at least one of the Heliconiina, *Heliconius
heurippa*, is a hybrid species, having characteristics inherited from the
local races of both *Heliconius cydno* and *H. melpomene* (Salazar
et al. 2005, Mavárez et al. 2006).

We here provide an updated database of wild-caught
interspecific *Heliconius* hybrids. In this butterfly genus, about
26% of species are known to hybridize (Mallet et al. 1998, Mallet 2005).

For many of these species, laboratory
hybrids have now been produced.  We have excluded any laboratory hybrids
from the database because we were interested here mainly in the potential for
natural hybrids.  However, the artificially produced hybrids are a useful
confirmation of the hybrid status of the wild-caught individuals. Several recent
studies have dealt with laboratory hybridization, the inheritance of colour
pattern, and hybrid viability and sterility between Heliconius
species (Jiggins & McMillan 1997, McMillan
et al. 1997, Naisbit et. al. 2002, 2003).  Gynandromorphs, presumably a
result of chimaeric development of separately fertilized zygotes, are relatively
common in hybridization experiments
between geographic races of  Heliconius
species.  This may merely be due to the greater ease of detection of gynandromorphs
in populations polymorphic for major colour pattern differences. 
Larry Gilbert (pers. comm.) obtained an interesting gyndandromorph
Heliconius cydno x H.
melpomene hybrid, perhaps the only one of its kind.

Several specimens are unique and may be simple
mutational variants, as opposed to hybrids.  These have been excluded from
our database as far as possible; for example, we here show a very
odd *Eueides* caught in the wild, and an aberrant
Heliconius charithonia produced
in an insectary. Other probable mutant specimens are shown here.

Between most pairs of species, hybrids
are very rare in nature. The only exceptions are *H. himera* and *H.
erato*, which hybridize wherever their ranges abut in contact zones.
In this pair of species, there is no inviability or sterility among the
hybrids, backcrosses, or F_2_ (McMillan et al. 1997). The species remain
distinct because of mate choice (which is about 5% "leaky"), and strong
ecological selection against hybrids.

In another good example, *Heliconius melpomene*
and *H. cydno* hybridize regularly (though at low frequency, maybe 1/1000
individuals are hybrids) throughout their joint range, and their distributions
overlap extensively throughout W. Ecuador, Andean Colombia and Venezuela, and
Central America. Here female hybrids have been found in the laboratory to be
sterile (Naisbit et. al. 2002, 2003), but wild hybrids are often backcross phenotypes,
showing that males backcross in the wild as well as in captivity. Once hybrids
have been produced in a local population, backcross phenotypes may survive at
high frequency for several generations. In a
collection of 103 *H. cydno* and *H. melpomene* made by Jesús
Mavárez in the botanic garden of San Cristobal, Táchira, Venezuela,
seven were putative backcross hybrids, even though such hybrids are rare elsewhere.
These two species are extremely closely related genetically, and the rarity
of hybrids is due to very strong mate discrimination (Jiggins et al. 2001).
Some mtDNA studies put *H. cydno* within the genealogy of *H. melpomene*;
i.e. *H. cydno* is little more than a clade of *Heliconius melpomene*
that has speciated,suggesting *H. melpomene* a paraphyletic remnant (Brower
1996).

An important conclusion that can be
drawn from this kind of data is that speciation doesn't suddenly lead
to a complete absence of gene flow. There may be several millions of
years after speciation during which genes may be exchanged between
newly-evolved, recognizably and ecologically distinct species. Given
that new species are able to maintain genetic differences in spite of
hybridization, interspecific gene flow between animal species could be
quite common, and may even contribute to heritability and genetic
variation within animal species, as has been shown for the Darwin's
finches by Grant & Grant (1992). There is very clear evidence in
the specimens we illustrate that wild hybrids between *H. melpomene*
and *H. cydno*, and between *H. erato* and *H. himera*,
backcross regularly. We are currently undertaking studies to
investigate whether significant gene flow occurs in some parts of the
genome between *Heliconius melpomene* and *H. cydno*, while
leaving other parts of the genome, affecting ecological and colour
pattern differences, intact.

Another conclusion is that reinforcement (adaptive
evolution of mate choice) may be more likely than previously realized. Reinforcement
is often seen as unlikely because the evolution of mating isolation has to race
against the breakdown of the genetic differences due to hybridization - the
latter will usually win. But, given that newly emerged species can stably maintain
their genetic differences in the face of gene flow, further mate choice should
be able to evolve adaptively to prevent the production of genetically inferior
hybrids.

References  
  
Bates, H.W. 1863. A Naturalist on the River Amazons. London: John Murray.

Beebe, Polymorphism in reared broods of Heliconius
butterflies from Surinam and Trinidad. Zoologica, N.Y. 40: 139-143

Brower, A.V.Z. 1996.
Parallel
race formation and the evolution of mimicry in *Heliconius* butterflies:
a phylogenetic hypothesis from mitochondrial DNA sequences. Evolution
50: 195-221.  
  
Grant P.R. & Grant B.R. 1992.
Hybridization
of bird species. Science 256: 193-197.  
  
Guillaumin, M. & Descimon, H. 1976, in: *Les Problèmes de l'Espèce
dans le Règne Animal*. Vol. 1. Eds: Bocquet, C., Génermont,
J., & Lamotte, M., Société zoologique de France, Paris, 129-201.  
  
Jiggins, C.D. & McMillan, W.O. 1997. The genetic basis of an adaptive radiation:
warning colour in two Heliconius
species. Proc. Roy. Soc. Lond. B  264: 1167-1175.

Jiggins, C.D., Naisbit, R.E., Coe, R.L. &
Mallet, J. 2001.  Reproductive isolation caused by colour pattern mimicry. 
Nature 411: 302-305.

Mallet, J. 2005. Hybridization as
an invasion of the genome. Trends in Ecology and Evolution 20: 229-237.

Mallet, J., McMillan, W.O. & Jiggins, C.D.
1998. Mimicry and warning color at the boundary between races and species. In:
Endless Forms: Species and Speciation. Eds: Howard, DJ & Berlocher, SH,
Oxford Univ. Press, New York, 390-403.

Mavárez, J., Salazar, C., Bermingham, E.,
Salcedo, C., Jiggins, C.D. & Linares, M. 2006. Speciation by hybridization
in *Heliconius* butterflies. Nature 441: 868-871.  
  
McMillan, W.O., Jiggins, C.D., & Mallet, J. 1997.
What initiates speciation in passion-vine butterflies?
Proc. Natl. Acad. Sci. USA 94: 8628-8633.

Naisbit, R.E., Jiggins, C.D., Linares, M., Salazar,
C. & Mallet, J. 2002. Hybrid sterility, Haldane's rule, and speciation in
*Heliconius cydno* and *H. melpomene.* Genetics 161: 1517-1526.  
  
Naisbit, R.E., Jiggins, C.D. & Mallet, J. 2003.
Mimicry: developmental genes that contribute to speciation. Evolution and Development
5(3): 269-280.

Salazar, C.A., Jiggins, C.D., Arias,
C.F., Tobler, A., Bermingham, E., & Linares, M. 2005. Hybrid incompatibility
is consistent with a hybrid origin of *Heliconius heurippa* Hewitson
from its close relatives*Heliconius cydno* Doubleday and *Heliconius
melpomene* Linnaeus. Journal of Evolutionary Biology 18: 247-256.

---

J. Mallet
home page   
**Last updated:** 18 September 2006
